# Novel approach to heritability detection suggests robustness to paternal genotype in a complex morphological trait

**DOI:** 10.1002/ece3.2932

**Published:** 2017-05-01

**Authors:** Max E. Winston, Andrea Thompson, Gabriel Trujillo, Andrew T. Burchill, Corrie S. Moreau

**Affiliations:** ^1^ Committee on Evolutionary BiologyUniversity of ChicagoChicagoILUSA; ^2^ Department of Science and EducationIntegrative Research CenterField Museum of Natural HistoryChicagoILUSA; ^3^ School of Life SciencesArizona State UniversityTempeAZUSA

**Keywords:** army ants, *Eciton burchellii*, geometric morphometrics, heritability, morphological plasticity

## Abstract

Heritable variation is essential for evolution by natural selection. In Neotropical army ants, the ecological role of a given species is linked intimately to the morphological variation within the sterile worker caste. Furthermore, the army ant *Eciton burchellii* is highly polyandrous, presenting a unique opportunity to explore heritability of morphological traits among related workers sharing the same colonial environment. In order to exploit the features of this organismal system, we generated a large genetic and morphological dataset and applied our new method that employs geometric morphometrics (GM) to detect the heritability of complex morphological traits. After validating our approach with an existing dataset of known heritability, we simulated our ability to detect heritable variation given our sampled genotypes, demonstrating the method can robustly recover heritable variation of small effect size. Using this method, we tested for genetic caste determination and heritable morphological variation using genetic and morphological data on 216 individuals of *E. burchellii*. Results reveal this ant lineage (1) has the highest mating frequency known in ants, (2) demonstrates no paternal genetic caste determination, and (3) suggests a lack of heritable morphological variation in this complex trait associated with paternal genotype. We recommend this method for leveraging the increased resolution of GM data to explore and understand heritable morphological variation in nonmodel organisms.

## 1 Introduction

Complex quantitative traits are ubiquitous in natural populations, and often mediate important aspects of organismal niche. Morphological traits tend to be more heritable than physiological and life history traits (Roff & Mousseau, 1987; Visscher, Hill, & Wray, 2008), with several well‐documented patterns of highly heritable craniofacial characteristics in vertebrates (Adams, 2011; Carson, 2006; Johannsdottir, Thorarinsson, Thordarson, & Magnusson, 2005; Postma, 2014), as well as numerous traits in insects (Roff, 1986; Schwander, Rosset, & Chapuisat, 2005). Geometric morphometrics (GM) are commonly used for the quantification of morphological variation because they provide better resolution than linear measurements, especially in exploratory analyses (Webster & Sheets, 2010; Zelditch, Swiderski, & Sheets, 2012). Although analytical solutions exist for applying quantitative genetics to linear measurements—such as the calculation of detectable effect size—these solutions do not generalize to GM due to the interdependence of shape data and the use of Procrustes transformation in shape analysis (Falconer & Mackay, 1996; Lande, 1979). Thus, researchers interested in the heritable component of morphological traits in nonmodel organisms would greatly benefit from new methods for heritability detection that harness the quantitative resolution of GM.

Neotropical army ants are obligate social predators where cooperation between hundreds of thousands of sterile workers is critical to the success of the colony (Franks, 1986). The Neotropical army ant *Eciton burchellii* is the premier example of this cooperation, containing colonies with several behaviorally, morphologically, and physiologically distinct sterile worker subcastes (Berghoff, 2003; Franks, Sendova‐Franks, & Anderson, 2001; Westwood, 1842) generated from known developmentally plastic pathways (Abouheif & Wray, 2012; Libbrecht et al., 2013). Although morphologically distinct, the worker subcastes demonstrate continuous variation in size and are delimited by size thresholds along this range (Franks, 1985). *Eciton burchellii* is highly polyandrous, with a single queen who mates multiply in a single interval and can produce millions of individuals over her lifespan from the stored sperm (Barth, Moritz, & Kraus, 2014; Kronauer, Berghoff, et al., 2006). Despite the fact studies have documented the role of developmental plasticity in generating the morphological diversity observed among worker subcastes (Abouheif & Wray, 2012; Jaffé, Kronauer, Kraus, Boomsma, & Moritz, 2007), it is unknown whether there is genotypic bias in the production of morphological traits. This question is critical to understanding the evolution of the ecologically important trait of head shape in workers of *E. burchellii* (Powell & Franks, 2006), since heritable variation is the primary material used by natural selection (Falconer, 1960). Furthermore, the mating system presents a unique opportunity to explore heritability of morphological traits among related workers in half‐sibling families that all share the same colonial environment.

The high rate of polyandry found in army ant lineages contributes to increased genetic diversity within a colony (Barth et al., 2014; Jaffé, Moritz, & Kraus, 2009; Jaffé et al., 2007; Kronauer, Johnson, & Boomsma, 2007; Kronauer, Schöning, & Boomsma, 2006). Several leading hypotheses to explain the large number of multiple matings revolve around the advantages of genetic diversity, which, for example, may increase resistance to a large variety of parasites (Hughes & Boomsma, 2006; Kronauer, Schöning, et al., 2006; Van Baalen & Beekman, 2006). Polyandry may also facilitate the evolution of task specialization, promoting division of labor within a colony by increasing morphological diversity among workers (Oldroyd & Fewell, 2007). This hypothesis is often considered in studies demonstrating a significant genetic basis to worker caste determination (Hughes, Sumner, Van Borm, & Boomsma, 2003; Jaffé et al., 2007; Keller, Sundström, & Chapuisat, 1997) and suggests that morphological traits themselves may be heritable. Thus, not only does the unique mating system of *E. burchellii* provide a unique opportunity to learn about heritability within a plastic system, but studies also suggest that the high mating frequency could be driven by natural selection on increased morphological diversity (Jaffé et al., 2007; Kronauer, Schöning, et al., 2006).

Existing quantitative genetic and GM methods offer an important foundation for exploring heritability in nonmodel organisms (Adams, 2011; Falconer & Mackay, 1996; Klingenberg, Leamy, & Cheverud, 2004). By pairing existing methods with simulations based on empirical data, our approach provides a standardized procedure for guiding experimental design and understanding detectable effect size in exploratory empirical work for nonmodel organisms. Furthermore, the use of dimensionality reduction in our likelihood framework improves detection of heritable variation along shared axes of variation, which may be expected in many organismal systems (Aubin‐Horth & Renn, 2009; McGuigan, Nishimura, Currey, Hurwit, & Cresko, 2010). For example, sensitivity to hormonal signaling during development—such as juvenile hormone in developing insects—may produce shared heritable variation between different genotypes (Nijhout, 2003; Nijhout & Wheeler, 1982; Zera, 2006).

Here, we present a new method employing a relatedness matrix and high‐dimensional GM data that can robustly recover heritable morphological variation among several half‐sibling groups and in the presence of strong nonheritable variation. The method generalizes to any system of related individuals and can be applied with any set of landmarks that are appropriate for GM analysis. Our method is novel in that it provides a simulation‐based solution for applying quantitative genetics to GM data, using a dimensionality reduction approach that explicitly searches for concerted heritable variation among half‐sibling groups. We demonstrate how the application of our method can be used in a nonmodel organism to address fundamental questions of evolution by investigating the mating frequency and heritability of morphological traits seen in the worker caste of *E. burchellii*.

## 2 Methods

### 2.1 Sample collection

Samples for the study were collected in June 2012 from the Area de Conservación Guanacaste (ACG), in northwestern Costa Rica. In total, we collected 216 individual sterile workers from three different colonies, which were sampled for genetic and morphological analyses. The 216 individuals were sampled by colony in the following breakdown: 48 from Colony 1 (C1), 72 from Colony 2 (C2), and 96 from Colony 3 (C3). Voucher specimens have been deposited in the biological collections of the Field Museum of Natural History (FMNH), Chicago, IL, USA.

### 2.2 Genotyping

Two sets of microsatellites were used for genotyping. First, we used three of eight highly polymorphic microsatellites previously isolated from *E. burchellii foreli* (Denny, Franks, & Edwards, 2004; Winston, Kronauer, & Moreau, 2016). The chosen highly polymorphic microsatellites were chosen due to amplification performance, in order to reduce the prevalence of null alleles. Second, we used 10 of 45 conserved microsatellite loci identified from a study of eight phylogenetically dispersed ant genomes (Butler, Siletti, Oxley, & Kronauer, 2014). Conserved microsatellite loci were selected in order to maximize polymorphism among the samples.

In order to preserve individuals for morphological analysis, we extracted DNA from the three right legs of each specimen. We then homogenized the legs with a Qiagen Tissue Lyser and used a DNeasy Blood & Tissue Animal tissue spin column protocol to extract and purify the DNA following the protocols outlined by Moreau (2014). The polymerase chain reaction (PCR) master mix was comprised of the following: 4 μl H_2_O, 2 μl BSA (100X), 0.875 μl MgCL_2_ (25 mmol/L), 1 μl buffer with MgCl_2_ (10X), 0.6 dNTPs, 0.4 μl forward and reverse primers (10 μmol/L), 0.15 μl Taq polymerase (5 U/μl), and 1 μl DNA template for a total reaction volume of 10.4 μl. We ran the reaction in a Bio‐Rad Peltier Thermal Cycler with the following parameters: An initial denaturation of 4 minutes at 95°C, then thirty‐five cycles of 30 s at 95°C, 30 s at 55°C, 45 s at 72°C, and a final extension at 72°C for 7 min. We genotyped the PCR reactions using an Applied Biosystems 3730xl DNA Analyzer sequencer. Allele calling and fragment sizing of chromatograms were performed using Geneious R7 software (Kearse et al., 2012). We then added a quality control step by having an independent party call alleles and cross‐validating these results.

### 2.3 Parentage inference

Parentage inference in *E. burchellii* is facilitated by haplodiploidy and the presence of only one queen in each colony (Rettenmeyer & Watkins, 1978). We assigned queen and male genotypes using COLONY (Jones & Wang, 2010), which implements full‐pedigree likelihood methods to simultaneously infer sibship and parentage. Unlike many other parentage inference programs, COLONY can accommodate and estimate genotyping error at each locus. For robust parentage inference, we created several subsetted datasets by subsampling both individuals and loci and compared inferred parentage across the datasets. Genotyping error and paternal genotype mismatches were minimal (Appendices S1–S3); thus, the paternal genotypes used in our analysis were inferred from the maximum‐likelihood estimation from the full dataset.

### 2.4 Workflow for detection of heritable morphological variation

In order to estimate whether there is heritable morphological variation in the castes of *E. burchellii* and the effect size of heritable variation we could detect given our data, we created a workflow linking simulated and empirical genotypic and GM data to a likelihood‐based method. This workflow is outlined in Table 1.

**Table 1:** A workflow for detecting heritable morphological variation with GM data

Step 1. Landmark selection
Homologous landmarks are chosen to capture the variation associated with trait(s) of interest, (section 2.5)
Step 2. Preliminary data collection
A geometric morphometric dataset of the selected landmarks is collected to estimate and parameterize variation [section 2.6, Figure 2]. It is critical that within‐sample measurement error in selected landmarks is negligible in comparison with between‐sample variation and that the samples included in the preliminary data collection encompass the phenotypic variation observed in the organism
Step 3. Simulations of heritable variation
Simulations are parameterized with the morphological variation estimated from the preliminary dataset (section 2.6, Figure 2) and are specific to the set of homologous landmarks chosen in Step 1. The nature of the heritable variation (i.e., number of landmarks affected, covariance of morphological variation of distinct genotypes) should be based on assumptions determined from the relevant literature (section 2.6). Several effect sizes of heritable variation should be chosen to find the edge of the detectable range by iteratively using the likelihood test defined in this paper (section 2.6, Tables 1, 2). Investigator will determine a range of attainable sample sizes for data collection and will also need to define a relatedness matrix for the tested individuals based on the organismal system (section 2.7)
Step 4. Data collection for heritability estimation
Given the detectable effect sizes of heritable morphological variation for the range of sample sizes used in the simulations (Step 3), the investigator should collect GM data for a sample size that matches the desired effect size (section 2.8)
Step 5. Testing data for heritable variation
Once GM data for all samples have been collected, these data can be used to assess heritable variation with our likelihood‐based method. This involves constructing a relatedness matrix in a similar fashion to Step 3, except based on the relationships attained from empirical genetic data. See R script for running code for the method in Supplementary Information (Appendix S6) and available on GitHub (https://github.com/mewinsto)

### 2.5 Morphometrics

**Figure 1:**
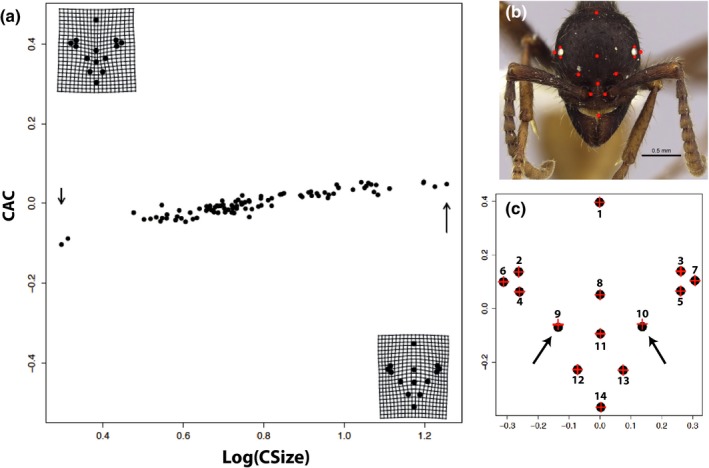
Visualizations of geometric morphometric landmarks, allometric deformation, and standard heritable deformation. (a) The allometric deformation (AD) is derived from the common allometric component (CAC) from 48 individuals and is visualized using thin‐plate splines in the top‐left and bottom‐right corners of the plot (arrowed individuals). (b) The 14 landmarks employed for this study demonstrated on a worker head case seen in red. (c) Reference form landmarks plotted with black dots, landmarks showing standard heritable deformation (HD1) plotted with red crosses to visualize effect size. Note that all landmarks (red crosses and black dots) are the same except for the two indicated with black arrows (landmarks 9 and 10), where the difference between the red crosses and black dots represents the effect size for HD1

We took both linear and geometric morphometric measurements on different body parts of sterile workers from all worker subcastes. Because back leg length (BLL) has been used in previous studies on *E. burchellii* as a proxy for body size (Powell & Franks, 2006), we included this measurement in our analysis. The rest of the measurements were taken on images of the head of the sterile workers using landmark‐based geometric morphometrics (GM), which are a set of methods for quantifying multidimensional shape data. Landmarks were chosen on the heads of the sterile workers for three reasons: (1) The heads demonstrate the most inter‐ and intraspecific morphological variation in comparison with other body parts, (2) head shape has been strongly associated with the behavioral ecology of the sterile workers (Powell & Franks, 2005), and (3) morphological variation in the head of the sterile workers has a strong link to the ecological dominance and niche of different *Eciton* species (Powell & Franks, 2006). Each specimen has 14 landmarks assigned to homologous points on the head case (Table 1: *Step* 1), as illustrated in Figure 1. Standard traditional morphometric measures such as head length (HL) and head width (HW) can also be calculated from distances between landmarks (Ward & Downie, 2005; Watkins, 1976).

We took images for GM analysis on the Photo Montage Leica Imaging Suite ver. 4.2 in the Collaborative Invertebrate Laboratory in the Field Museum of Natural History in Chicago, IL. We standardized orientation of the head so that the bilaterally symmetric plane between the clypeus and the occiput was orthogonal to the imaging direction in all specimens. The auto‐montage synthesized between 8 and 50 images for a single composite image for each specimen. We processed and digitized all images in ImageJ (Schneider, Rasband, & Eliceiri, 2012).

Morphometric analysis was primarily performed using the *geomorph* and *shapes* packages in R, as well as the IMP7 package (Adams & Otarola‐Castillo, 2013; Dryden & Mardia, 1998; Webster & Sheets, 2010). Analyses consisted of principal components analysis (PCA), modeling of static allometric curves, and calculation of morphological disparity. As generalized Procrustes analysis removes difference in sizes by scaling the landmark configurations by centroid size—the square root of the summed distances of all the landmarks to the centroid—the Procrustes‐fit shape variation can then be modeled against centroid size to model static allometry. Custom R scripts for each analysis mentioned above can be found in the supplementary information (Appendices S6, S7, and on GitHub: https://github.com/mewinsto).

### 2.6 Data simulation

In order to evaluate our ability to detect heritable morphological variation in a system with abundant nonheritable variation (Jaffé et al., 2007), we took an empirically based simulation approach using morphological data from 48 specimens to define traits from the measurements, and the static allometry (Table 1: *Step* 2). Detection of the heritable variation would take place within this parameterized allometric variation with additional noise to simulate measurement error (Appendix S8).

Specifically, after applying Procrustes analysis using the *geomorph* package to the set of 48 specimens, the *plotAllometry* function was then applied to the Procrustes‐fitted individuals, generating a multidimensional set of allometry curves known as the common allometric component (CAC) (Adams & Otarola‐Castillo, 2013). Using the predicted shapes from the generated allometry curves and the known centroid sizes of the individuals, linear models were then constructed for each individual landmark with the *stats* package, effectively parameterizing the static allometry for data simulations. This is defined as the allometric deformation (AD).

Because data collection of morphological characters will always introduce measurement error, a necessary component of modeling detection of heritable variation is noise. As determined by previous empirical error analysis of technical replicates, there is no evidence within our morphological measurements of anisotropic error (Appendix S13). Thus, we modeled our noise using the *rnorm* function from the *stats* package, with a mean of zero and a landmark‐specific standard deviation from the allometry‐removed empirical dataset. In the following work, this is defined as the standard noise deformation (ND1).

Parameterizing the heritable morphological variation is difficult as there are many different interpretations and models for heritable morphological traits (Aubin‐Horth & Renn, 2009; Nijhout & German, 2012; Nijhout & Wheeler, 1996). In order to keep the simulations to a manageable number of treatments, we ran two separate data simulations, the first of which aimed at understanding the necessary effect size of the heritable morphological variation for detection, and the second of which aimed at assessing our ability to detect different types of heritable variation within a sample of a greater number of patrilines.

In our first data simulation (Table 1: *Step 3*; Table 2), the treatment was a simple deformation of a given effect size applied to two landmarks for those individuals sharing one patriline, while the remaining individuals fathered with a second patriline were designated the reference form (Figure 1). We defined this as the standard heritable deformation (HD1). In order to assess our ability to detect this heritable variation, we ran this simulation under four treatments: *Treatment* 1 (T1) was the standard treatment, *Treatment* 2 (T2) was the same noise (ND1) and allometric (AD) deformations but with double the effect size of the heritable variation (HD2), *Treatment* 3 (T3) was HD1 and AD, but with double the noise (ND2), and *Treatment* 4 was ND1 and AD, but with half the heritable variation (HD3). Finally, each treatment was put in Procrustes superimposition using the *gpagen* function from the *geomorph* package, followed by principal components analysis of this shape variation with the *plotTangentSpace* from the *geomorph* package (Adams & Otarola‐Castillo, 2013).

**Table 2:** Treatment definitions for first data simulation

Data simulation 1		
Treatment	Heritable deformation	Noise deformation
T1	Standard (HD1)	Standard (ND1)
T2	Double (HD2)	Standard (ND1)
T3	Standard (HD1)	Double (ND2)
T4	Half (HD3)	Standard (ND1)

In our second data simulation (Table 1: *Step 3*; Table 3), we focused on the robustness of the model to higher mating frequencies and different deformation types. To accomplish this, we implemented two types of heritable morphological variation at two different strengths, across four mating frequencies (3, 5, 10, 20). First, the most straightforward treatment was a simple deformation of a given effect size applied to two landmarks, where the strength of the maximum effect size was independent of the mating frequency, defined as the independent heritable deformation (HD5). Second, the effect size of the variation was slightly increased with increases in mating frequency, defined as the nonindependent heritable deformation (HD6). These treatments can then be applied to the two landmarks in different directions, orthogonal (o) and parallel (p), creating four total treatments for the data simulations (T5o, T5p, T6o, T6p). Matching our first set of simulations, each treatment was then put in Procrustes superimposition using the *gpagen* function from the *geomorph* package, followed by principal components analysis of this shape variation with the *plotTangentSpace* from the *geomorph* package (Adams & Otarola‐Castillo, 2013).

**Table 3:** Treatment definitions for second data simulation

Data simulation 2			
Treatment	Heritable deformation	Noise deformation	Orientation
T5o	Independent (HD5)	Standard (ND1)	Orthogonal
T5p	Independent (HD5)	Standard (ND1)	Parallel
T6o	Nonindependent (HD6)	Standard (ND1)	Orthogonal
T6p	Nonindependent (HD6)	Standard (ND1)	Parallel

In summary, for any randomly generated set of *n* individuals—where centroid sizes are parameterized by the empirical set of 48 individuals—data can be simulated by simply adding the three types of deformation (AD, ND, and HD) to the mean shape for each individual (Appendix S8). The custom R script written to perform the data simulation is included in the Supplementary Information (Appendix S6 and available on GitHub: https://github.com/mewinsto). All datasets were created for 100 individuals to match given sample sizes of the empirical dataset and then replicated under all treatments 100 times to test statistical power.

### 2.7 Maximum‐likelihood method for detecting heritable morphological variation

In order to detect significant heritable morphological variation in both simulated and empirical datasets, we created and utilized a maximum‐likelihood (ML) approach (Table 1: *Step* 5) based on the probability model defined in Equations (1), (2), (3):

(1) $$\text{PC}∼\text{MVN}(\mu_{A},\;\left[\text{VCV}\right]\sigma^{2})$$

(2) $$\mu_{A}=\beta \left(\text{size}\right)+\mu_{0}$$

(3) $$\left[\text{VCV}\right]=\left[K\right]h^{2}+\left[I\right]\left(1-h^{2}\right)$$

where the principal component scores (PCs) are distributed as a multivariate normal (MVN) with vector of means equal to an allometric model of mu (μ_A_) and variance equal to an among‐observation variance‐covariance matrix ([VCV]) multiplied by a variance coefficient (eq. (1)). The allometric model (μ_A_) is detailed in Equation (2), defined simply as the centroid size scaled by an allometric coefficient (β) with a mean value of mu (μ_0_). Lastly, the expected variance‐covariance matrix ([VCV]) under a heritable trait can be modeled as the proportion of variance expected to covary by a relatedness matrix ([K]), times the heritability (*h* ^2^), added to the nonheritable proportion of variance (1 − *h* ^2^) expected to vary independently as defined by the identity matrix ([I]). The partitioning of the variance‐covariance matrix follows the common mixed‐model analysis in quantitative genetics (Falconer & Mackay, 1996; Speed, Hemani, Johnson, & Balding, 2012), and further notes on derivation can be found in the supplementary information (Appendix S12). Due to the facts that *E. burchellii* are haplodiploid, have a single mated queen (Kronauer, Berghoff, et al., 2006), and tested individuals were sterile workers, the relatedness matrix ([K]) was constructed using relatedness coefficients of 0.75 for individuals with the same father, 0.25 for individuals with different fathers, and 0 for individuals from different colonies.

To evaluate our ability to detect heritable morphological variation, we applied a likelihood ratio test (LRT) using the R package “*bbmle”* (Bolker, 2016), with a null hypothesis that the complex trait was not heritable at all (H_0_: *h* ^2^ **= 0), and an alternative that there was some heritable component to the variation (H_A_: *h* ^2^ > 0). Specifically, the LRT is illustrated in *Equation* 5, where the null hypothesis maximizes over all nuisance parameters (η) and constrains heritability to zero, whereas the alternative hypothesis maximizes both the nuisance parameters (η) and the heritability parameter (*h* ^2^):

(4) $$L_{0}=\max\left[\eta \right]\;\;L\left(h^{2}=0,\eta \right)$$

(5) $$L_{A}=\max\left[h^{2},\eta \right]\;\;L\left(h^{2},\eta \right)$$

Standard to the LRT, the test statistic (λ) is then calculated and should be distributed as a chi‐squared with a mixture of one degree of freedom (λ ~ *X*_1_^2^) and zero degrees of freedom (λ ~ *X*_0_^2^) as the heritability statistic cannot be negative (Pinhero & Bates, 2000; Self & Liang, 1987). Following this, because the LRT was applied to the top four PC scores, the Bonferroni‐corrected significance value (α) is 0.0125. Although we chose to test four PCs due to the nature of our empirical dataset—in particular the distribution of our eigenvalues—any number of PCs can be used for our approach, so long as the significance value (α) is adjusted accordingly. However, investigators should avoid using all the PCs, as it reduces the relative power of this method. The custom R script employed the R package “*mvtnorm”* for generation of null data.

### 2.8 Testing empirical data

Testing the generated genotypic and morphological data for genotypic bias and heritable variation in caste was accomplished using a number of methods. We stress that the application of our approach in *E. burchellii* leverages the known genetic variation within a single colonial environment (C3) due to high rates of polyandry (Barth et al., 2014; Jaffé et al., 2007, 2009; Kronauer, Berghoff, et al., 2006; Kronauer et al., 2007)—not between colonies—creating ideal conditions for testing for heritable variation (Falconer & Mackay, 1996). First, tests of genotypic bias in caste determination with two proxies for body size (BLL and centroid size) used an ANOVA among half‐sibling families of related workers. Second, testing for heritable variation followed the tests from the simulations for greater interpretability, which included permutational significance tests of canonical correlation analysis (CCA) and the ML approach on the top four PCs. Furthermore, to confirm the efficacy of our novel method, a published GM dataset of *Plethodon* salamander hatchlings with verified heritable morphological variation (Adams, 2011) was tested with our ML approach.

## 3 Results

### 3.1 Mating frequency

**Figure 2:**
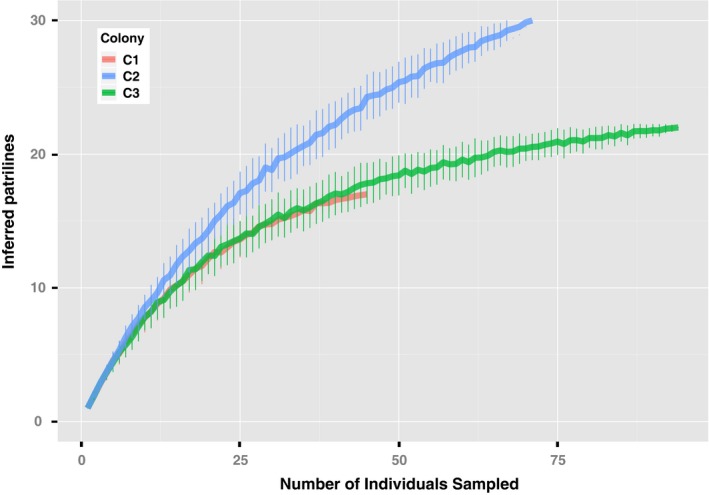
Patriline accumulation curves for all 216 genotyped individuals. Rarefaction accomplished using standard bootstrapping procedures for all three colonies, each of different sample size: 48 individuals genotyped for C1 (red), 72 individuals genotyped for C2 (blue), 96 individuals genotyped for C3 (green)

Mating frequency for all three *E. burchellii* colonies was estimated using COLONY. Despite continued accumulation of patrilines with increased sampling (Figure 2), the inferred number of patrilines was steady across colonies with increased number of microsatellite loci (Appendix S1). This finding is consistent with the fact that COLONY is conservative in parentage assignment by accounting for genotyping errors (Jones & Wang, 2010). Mean estimated error rates and standard deviation (σ) from parentage inference across all microsatellites and COLONY runs were 0.0371 for Colony 2 (σ = 0.0268), 0.0388 for Colony 3 (σ = 0.0165), and 0.0539 for Colony 1 (σ = 0.0295). Generally, mean error rates were higher in COLONY runs using a larger number of microsatellites; however, increases in error rates remain relatively low (Appendix S2). Across all COLONY runs, the mean error rate for all thirteen microsatellites was under 5% [μ = 0.045, σ = 0.015, *N* = 13]. Distributions for estimated error rates by microsatellite can be found in Appendix S3.

Generally, increased sampling of individuals led to an increase in observed mating frequency, suggesting that estimates of the true mating frequency are conservative (Figure 2). Nonetheless, the estimates for observed mating frequency in *E. burchellii parvispinum* [31 for C2 (*n = *72), 25 for C3 (*n = *96), and 17 for C1 (*n = *48)] are higher than previous estimates of 13–25 matings (Jaffé et al., 2007). Less conservative estimates based on rarefaction of individuals and a Chao1 estimator range from 46 for Colony 2, to 39 for Colony 3, and to 31 for Colony 1.

### 3.2 Morphological variation

Due to the multidimensionality of the GM data, PCA is an appropriate ordination method to explore morphological variation (Mitteroecker & Gunz, 2009). For example, the static allometry resulting from a developmentally plastic pathway is easily demonstrated by plotting centroid size against the first principal component (Appendix S9). As PCA of Procrustes‐fit GM data contains no information of centroid size, this relationship shows that the majority of variation among individuals (55.1%) results from static allometry associated with morphological plasticity. The static allometry produced by this morphological plasticity defines the differences between worker subcastes and is likely mediated by developmental pathways involving hormonal morphogens (Abouheif & Wray, 2012). For a more direct estimation of allometry, the CAC can be calculated using the *geomorph* package (Figure 1a), which explicitly models the variation by worker size (Mitteroecker, Gunz, Bernhard, Schaefer, & Bookstein, 2004).

### 3.3 Data simulation I

**Figure 3:**
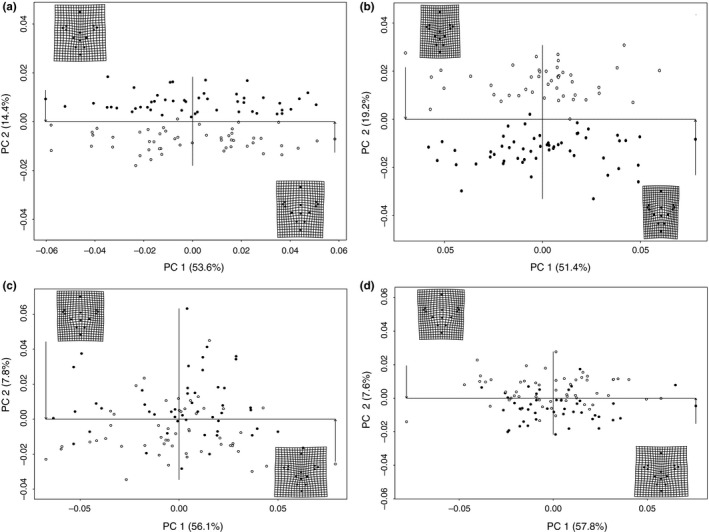
(a–d) Principal component plots for each treatment from data simulation 1. Treatments are labeled as following: T1 (a), T2 (b), T3 (c), T4 (d). Black and white dots denote individuals of the two different patrilines, which separate on PC2 to different degrees among the different treatments. See Figure 4 for detection capability based on these scores. Scores on PC1 are highly correlated with AD shape change, with the most extreme values on PC1 illustrated in the two thin‐plate splines for each plot

Using the common allometric component (CAC) derived from centroid size and from empirical morphological data (Figure 1a), we parameterized the static allometry and replicated this deformation (AD) for 100 individuals over 100 replicates. For each of these replicates, the plastic static allometry could be represented on a single PC axis (Appendix S10). Combining the allometric deformation (AD) with noise deformation (ND) and heritable deformation (HD) as described by our four treatments (T1, T2, T3, T4), we generated four datasets with 100 replicates each. Performing PCA on the replicates from each of the treatments demonstrated visible segregation for some of the treatments (Figure 3) and allowed for the construction of test statistics (eqs. (1), (2), (3)) for assessing detection power.

**Figure 4:**
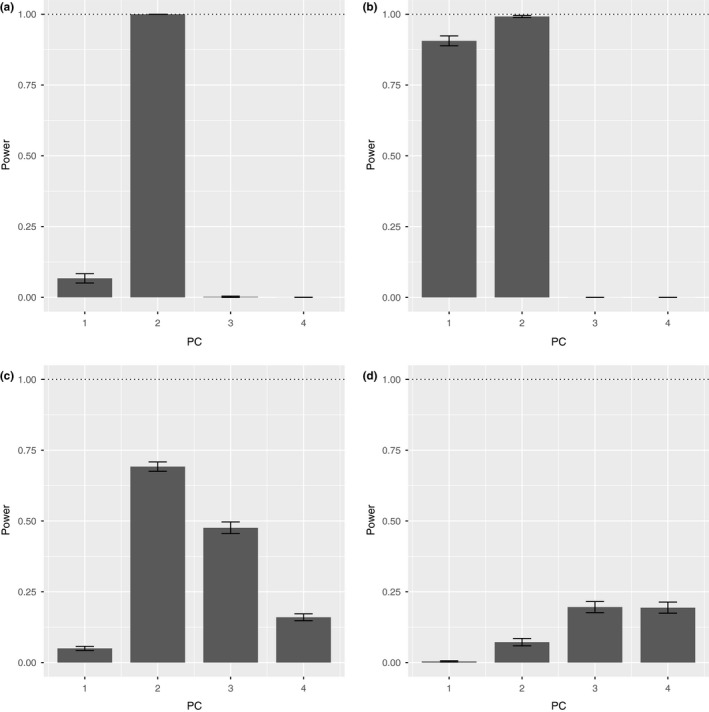
Statistical power for detection of heritable variation in data simulation 1. Plots demonstrate the number of 100 replicates recovered as statistically significant using each of the four principal components (PC1–PC4) for each treatment from data simulation 1. Error bars represent standard error. Treatments are labeled as following: T1 (a), T2 (b), T3 (c), T4 (d). Note the strong statistical power for detection of heritable variation with PC2 for T1 and T2, with the dotted line as the maximum statistical power (100%)

Assessing statistical power of detecting heritable variation was accomplished by recording the fraction of replicates able to recover a significant, the Bonferroni‐corrected LRT value (Figure 4). Generally, PC2 offered the highest statistical power for detection (*T*1* *=* *1.0 [*SE *=* *0.0], *T*2* *=* *0.992 [*SE *=* *0.004], *T*3* *=* *0.692 [*SE *=* *0.017], *T*4* *=* *0.072 [*SE *=* *0.013]), with PC1 offering statistical power only under the treatment (T2) with double effect size (*T*1* *=* *0.067 [*SE *=* *0.017], *T*2* *=* *0.906 [*SE *=* *0.017], *T*3* *=* *0.05 [*SE *=* *0.007], *T*4* *=* *0.004 [*SE *=* *0.002]). Statistical power of detection using PC3 (*T*1* *=* *0.002 [*SE *< 0.001], *T*2* *=* *0 [*SE *=* *0.0], *T*3* *=* *0.476 [*SE *=* *0.021], *T*4* *=* *0.196 [*SE *=* *0.020]) and PC4 (*T*1* *=* *0 [*SE *=* *0.0], *T*2* *=* *0 [*SE *=* *0.0], *T*3* *=* *0.160 [*SE *=* *0.012], *T*4* *=* *0.194 [*SE *=* *0.020]) for varied with treatment, but never reached higher than 50%.

### 3.4 Data simulation II

Creation of replicates for the second data simulation matched the first simulation, except that there were four treatments (T5o, T5p, T6o, and T6p) and four mating frequencies (3, 5, 10, and 20), resulting in a total of 16 datasets, each with 100 replicates of 100 individuals. Performing PCA on the replicates from each of the treatments demonstrated visible segregation for some of the treatments (Appendix S11) and allowed for the computation of test statistics (eqs. (1), (2), (3)) for assessing detection power.

**Figure 5:**
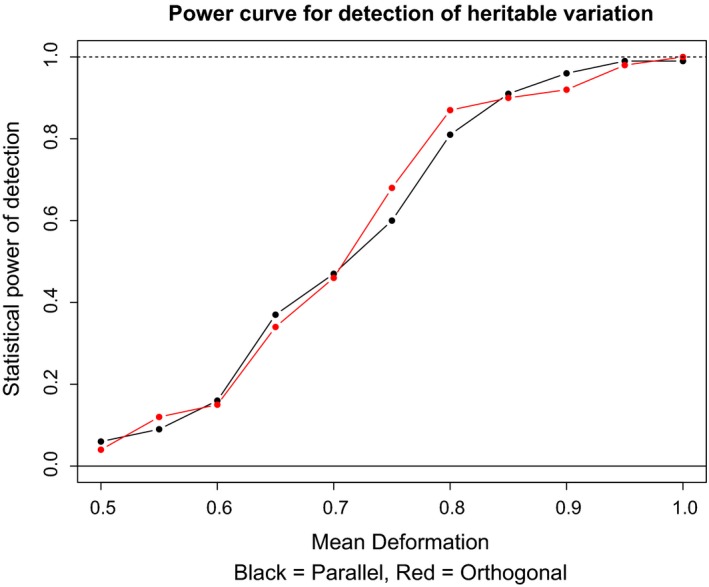
Statistical power for detection of heritable variation using PC2 in data simulation 2. Plot demonstrates the number of 100 replicates recovered as statistically significant using PC2, with the dotted line as the maximum (100/100). *X*‐axis is the mean deformation used in each set of simulations (replicate), with the standard effect size (HD1) as the largest value of 1.0. Detection of PC1, PC3, and PC4 for all treatments was 0 or below 0.02

Assessment of statistical power in the second data simulation was performed in the same manner as the first. For all 16 datasets, detection of heritable variation using PC2 was 1.0 or 0.99. Conversely, detection of PC1, PC3, and PC4 was 0 or below 0.02. In order to understand the rate at which statistical power to detect heritable variation decreased, we utilized a ten‐increment stepwise decrease in the variation, with the mean effect between the standard heritable variation (HD5), and half this value with the same noise deformation (ND1). Results demonstrate that statistical power to detect heritable variation decays between values of less than the standard heritable variation (HD5), but greater than half the standard heritable variation (Figure 5).

### 3.5 Method validation on Plethodon hatchling dataset

A dataset containing 282 hatchlings from 44 families of *Plethodon cinereus* salamander hatchlings with verified heritability (Adams, 2011) was tested with our heritability detection method. To mirror testing of our own data, we used the first four PCs of the Procrustes‐fit GM data for heritability detection, finding three of the four PCs as demonstrating highly significant heritable variation (PC1: *p* = 5.39e−11, PC2: *p* = 2.65e−05, *PC*3*: p = *0.814, PC4: *p* = 0.005) at a Bonferroni‐corrected significance threshold (α *= *0.0125). Estimated heritability values of morphological variation described by the three significant PCs ranged between 0.41 and 0.72 (PC1: *h* ^2^ *= *0.72, PC2: *h* ^*2*^ = 0.41, PC4: *h* ^2^ = 0.56).

### 3.6 Testing for genotypic bias in army ant caste determination

**Figure 6:**
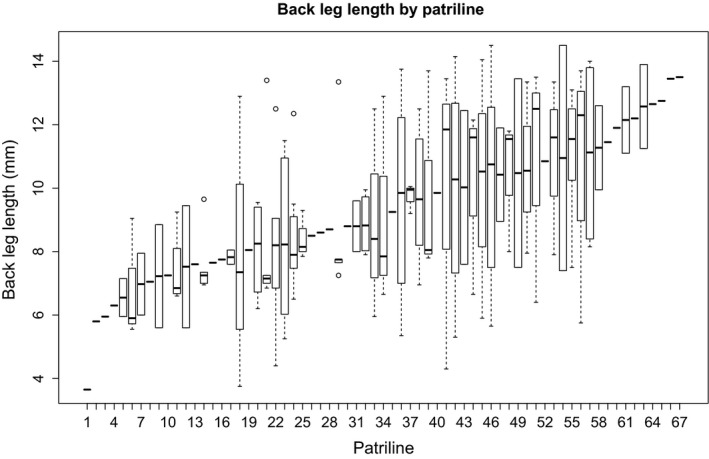
Boxplot of ranges of back leg length for all 73 patrilines from all three colonies. Sample sizes for each patriline range from singletons (indicated by bold dash) to 12 individuals (box and whiskers), and patrilines have been ordered by mean back leg length. Results demonstrated no significant caste bias based on paternal genotype

Because our study took measurements on two independent proxies for body size, tests for genotypic bias in caste determination used each of the proxies separately. First, for the 96 individuals that had GM measurements, we used centroid size as a proxy for caste. Under an ANOVA among patrilines, we found no significant effect of patriline on centroid size (*F* = 0.997, *p* = 0.32), with the mean ML estimate for effect size being 0.009. Second, with the measurements of back leg length from all 216 individuals genotyped (Figure 6), we found no significant effect of patriline (*F* = 0.949; *p* = 0.58), with the mean ML estimate for effect size being 1.19 mm. Simulations of detectable effect size for the ANOVA analysis of centroid size (Appendix S4) show a mean effect centroid size of 0.018 necessary for likely detection (>50% replicates) given our sample size (*n* = 96) and centroid size variation. Simulations of detectable effect size for the ANOVA analysis of back leg length (Appendix S4) show a mean effect size for back leg length of 1.4 mm necessary for detection (>50% replicates) given our sample size (*n* = 216) and back leg length variation.

### 3.7 Empirical testing for heritable morphological variation

Using the 96 individuals from C3 with GM measurements, we used a LRT to test the hypothesis that the heritability (*h* ^2^) was greater than zero (eq.** (5)). Testing each principal component (PC1–PC4), improvements in log‐likelihood under the alternative hypothesis were negligible in comparison with log‐likelihoods under the null hypothesis (λ: *PC*1: 0.61, *PC*2: 0, *PC*3: 0, *PC*4: 1.67), resulting in four heritability estimates (*PC*1: *h* ^2^ = 0.26 [*SE* = 0.36], *PC*2: *h* ^2^ = 0 [*SE* = 0], *PC*3: *h* ^2^ = 0 [*SE* = 0], *PC*4: *h* ^2^ = 0.45 [*SE* = 0.54],) with insignificant *p*‐values under a *X*_0.5_^2^ distribution (*PC*1: *p* = 0.23, *PC*2: *p* = 1.0, *PC*3: *p* = 1.0, *PC*4: *p* = 0.09). CCA permutational significance tests of partial warp scores and paternal genotype were also used to test for significance of heritable morphological variation, which resulted in zero significant canonical correlation coefficients (*p* = 0.682, 999 permutations).

## 4 Discussion

We do not find any significant heritable morphological variation in *E. burchellii* associated with the paternal genotype, despite the demonstrated ability of our novel method to detect heritable variation in several simulations and the empirically validated salamander hatchlings dataset (Figures 4, 5). Our simulations suggest that heritable morphological variation in very modest effect size—such as the minor change (0.01) of landmarks as seen in Figure 1c—should be detectable (*p* > 0.98) given the sample size and resolution provided by geometric morphometric data (Figures 4, 5, Appendix S11). In particular, results from the second data simulation suggest that the standard effect size (HD5 and HD6) should be easily detectable and robust to a variety of mating frequencies and directions (Figure 5). Therefore, from these simulations and our empirical testing, it is likely that if there is any heritable morphological variation relating to paternal genotype, it is either of negligible effect size or inherited by more complex mechanisms that cannot be captured with narrow‐sense heritability (*h* ^2^). The absence of this heritable variation in our empirical dataset implies a weak capacity for worker head shape, which is considered a proxy for subcaste and ecological role, to respond to selection on paternal genotype within this population.

We found higher rates of mating frequency demonstrated in this study compared to previous work on *E. burchellii* (Denny, Franks, Powell, & Edwards, 2004; Jaffé et al., 2007; Kronauer, Schöning, et al., 2006, Kronauer, Berghoff, et al., 2006). This is unlikely to reflect genotyping errors because of the robustness of mating frequency to the number of microsatellites used for parentage inference in this study and stringent quality control measures (Appendix S1). Factors contributing to intraspecific variation in mating frequency are unknown in Neotropical army ants, but ecological dynamics such as population density could have an important effect on mating frequency. Previous work on mating frequency was conducted in central Panamá on a different subspecies, *E. burchellii foreli*, so it is also possible that the subspecific taxonomy represents substantial genetic differences between the groups that may be accompanied by different mating dynamics. Although previous work has found significant genetic caste determination in *E. burchellii* (Kronauer, Berghoff, et al., 2006), this work is debated due to the confounding effects of patriline shifting (Wiernasz & Cole, 2010), an effect to which our work is also susceptible. As the observed genotypic bias found by Jaffé et al. (2007) was of very small effect size and tested with a larger sample size, it is not surprising that we failed to recover a similar, weak genotypic bias in caste determination among our samples, if their result is in fact valid (Kronauer, Berghoff, et al., 2006).

Along with the demonstrated weak capacity for selective response on paternal genotype, the high rates of mating frequency in *E. burchellii* have relevant consequences for morphological evolution of the sterile worker phenotypes on generational time scales. Specifically, as any individual male can only mate once and has a very low chance of contributing any genetic material to a successful reproductive (Kronauer, 2009), response to selection on heritable, patrigenic morphological variation among sterile workers is effectively absent. Of course, this study cannot determine the heritable morphological variation via maternal genotype, as the limitation of a single queen per colony prevents our ability to parse environmental and genetic components of morphological variation without experimental manipulation (Falconer, 1960). Nonetheless, given what is known about the role of hormonal signaling and developmental plasticity in social insects, it is more likely that heritable morphological variation in the worker castes passes through the queen rather than the males (Libbrecht et al., 2013; Simola et al., 2013; Zera, 2006). The lack of heritable morphological variation found in our study only strengthens this hypothesis.

Despite the fact that we recovered heritable variation in our positive control using the *Plethodon* dataset, mating system differences between the *Plethodon* salamanders and *Eciton* army ants may make detection of heritable variation in our army ant dataset more difficult than in the salamanders dataset. The most evident difference between the mating systems is that heritability in the *Plethodon* salamanders was calculated by estimating relatedness of hatchlings from the same clutch rather than direct genotyping (Adams, 2011). While this avoids the potential issue of error in the inference of paternal genotype, it also confounds other environmental factors shared among clutches, which may be interpreted as heritable variation. The relatedness matrix in our study was constructed by whether paternal and maternal genotype was shared by individual workers, rather than using an estimated genetic distance. Although this may have offered lower resolution than using genetic distance, it is still more genetically accurate than the methodology in the *Plethodon* dataset, by the use of direct genotyping. Lastly, the high rates of polyandry may have additional consequences, particularly if the effect sizes of heritable variation between paternal genotypes differ widely. Specifically, if paternal genotypes with large effect sizes are not sampled sufficiently, the power to detect heritable variation will be hindered.

High rates of polyandry can generate intense sexual conflict from divergent genetic interests of males and females (Chapman, Arnqvist, Bangham, & Rowe, 2003; Mank, Wedell, & Hosken, 2013). One relevant explanation to resolve the paradox between the observed mating dynamics in eusocial Hymenoptera and the theoretical expectations of sexual conflict with high variance in male reproductive success is parent‐of‐origin genomic imprinting (Drewell, Lo, Oxley, & Oldroyd, 2012; Gregg, Zhang, Butler, & Haig, 2010; Haig, 2000; Reik & Walter, 2001). Genomic imprinting is a common result of antagonism between the parents over growth and provisioning (Mank et al., 2013), and already known to be responsible for sex determination in a haplodiploid system (Verhulst, Beukeboom, & van de Zande, 2010). While the prevalence of imprinting across eusocial species still needs to be tested, epigenetic modification of specific growth‐related loci offers a clear mechanism for resolving sexual conflict in mating systems with a multiply mated single queen (Alvarado, Rajakumar, Abouheif, & Szyf, 2015; Drewell et al., 2012). Moreover, if in fact maternal silencing of patrigenes is a common response to sexual conflict, this offers a convenient mechanism that could have been co‐opted by queens to control their extended phenotype of sterile workers in highly eusocial organisms (Haig, 2000; Reik & Walter, 2001). In this case, we would not expect to detect any heritable morphological variation derived from the fathers, as patrigenic loci would be silenced.

We suggest that our finding of no detectable heritable morphological variation within our sample is the result of stronger environmental determinants and maternally transmitted genetic variation, which are responsible for the vast majority of morphological variation. While it is possible that morphological variation in this complex quantitative trait has been decoupled from genetic variation, the literature suggests that the trait is likely to be controlled maternally (Libbrecht et al., 2013; Nijhout, 2003; Nijhout & Wheeler, 1982; Zera, 2006). As simulated data did not incorporate structured sources of morphological variation outside of heritable variation and known plastic variation, it is possible that empirical data may deviate from simulation models. However, with no available evidence that unobserved environmental heterogeneity—such as worker diet during development—is responsible for structured morphological variation in Neotropical army ants, we avoided adding complexity to simulations. Lastly, although our data cannot address whether the maternal influence is a result of maternal genotype or environment, future studies may be able to tease these factors apart by comparing age‐standardized cohorts from several queens. More directly, a common garden experiment could clarify maternal influence on worker morphology by exchanging developing individuals between colonies and measuring phenotypic difference.

In summary, we present a new approach that leverages the resolution of GM for detecting heritable variation in nonmodel organisms. As evidenced by the application of our method to the empirical data in *E. burchellii* and *Plethodon cinereus*, our method can recover heritable variation of very modest effect size (Figure 1c), as well as properly estimate the detectable effect size for a GM dataset. Using dimensionality reduction, our approach offers a roadmap for estimating detectable effect size of concerted heritable variation with GM data and is a useful advance for the interpretation of morphological variation in nonmodel organisms. Specifically, although detectable effect size can also be calculated by simulations with some existing methods (Adams, 2011), our method allows for a more precise detection of shared variation through the use of dimensionality reduction, which we may expect in certain systems with demonstrated allometry like ants. Additionally, by parameterizing and estimating the sources of nonheritable variation—such as measurement error and morphological plasticity as accomplished in this study—our method defines what is possible to detect within a given GM dataset. Although we have taken advantage of the highly polyandrous and haplodiploid mating system in *E. burchellii* to exhibit the features of our method, we stress that it can be employed in any organism with known relatedness among groups. Given the many advantages of applying GM to nonmodel organisms, our simulation‐based approach for assessing the statistical power for detecting a range of effect sizes is highly valuable to researchers interested in using GM for quantitative genetics.

## Authors’ Contributions

AT generated genetic data, called microsatellites, and inferred patrilines. GT generated genetic data and inferred patrilines. AB generated genetic data and inferred patrilines. MEW designed experiment, created the method, ran the simulations, took GM measurements, performed GM analysis, and wrote manuscript. CSM designed experiment and was a major contributor in writing the manuscript. All authors read and approved the final manuscript.

## Conflict of Interests

The authors declare that they have no competing interests.

## Ethics Approval

The research in this manuscript follows all ethical guidelines for scientific research.

## Availability of Data and Materials

All microsatellite and GM data will be published on Dryad with acceptance of this manuscript.
