# Supplementary material for: Novel approach to heritability detection suggests robustness to paternal genotype in a complex morphological trait
Source: Ecol Evol. 2017 May 1;7(12):4179–91. doi: 10.1002/ece3.2932 (PMC5478049; doi:10.1002/ece3.2932)
Supplement: Supplementary file 1 [file ECE3-7-4179-s001.docx]

**Supplementary Information**

**SI. 1. Number of patrilines inferred for each colony by number of microsatellite loci included.** Solid lines for Colony 100 (red), Colony 102 (green), Colony 105 (blue) indicate number of patrilines inferred, while dotted lines infer total number of individuals sampled for each colony (maximum possible patrilines). Note the robustness of the number of patrilines inferred with respect to the number of microsatellite loci used for patriline inference.

**SI. 2. Error rates for all microsatellites for Colony 100 (a), Colony 102 (b), & Colony 105 (c).** Microsatellite color and IDs are defined in legend (right). Error rates were estimated by the parentage inference software COLONY. Note the reduced conflict in error rates when using 11 microsatellite loci.

**SI. 3a. Distributions of error rates for all microsatellites.** Microsatellites with mean error rates over 0.05 were removed for patriline identification.

| **Microsatellite** | **Mean (μ)** | **SD (σ)** | **N** |
| --- | --- | --- | --- |
| *Ant11893* | 0.044 | 0.022 | 15 |
| *Ant3648* | 0.052 | 0.012 | 16 |
| *Ant4155* | 0.036 | 0.024 | 16 |
| *Ant1368* | 0.048 | 0.031 | 11 |
| *Ant2341* | 0.059 | 0.024 | 11 |
| *Ant859* | 0.048 | 0.023 | 15 |
| *Ant575* | 0.022 | 0.010 | 16 |
| *Ant10878* | 0.031 | 0.016 | 16 |
| *Ant1343* | 0.056 | 0.050 | 10 |
| *Ant9218* | 0.020 | 0.008 | 16 |
| *Eb25* | 0.036 | 0.020 | 16 |
| *Eb10* | 0.068 | 0.010 | 11 |
| *Eb42* | 0.061 | 0.021 | 15 |

**SI. 3b. Table of error rates for all thirteen microsatellites used in this study.** Mean (μ) indicates mean error rate across all COLONY runs inferring parentage with that microsatellite (N), including information on standard deviation (σ).

**SI. 4. Simulation of detectable effect size of genetic caste determination.** Dashed line (horizontal) represents p-value of 0.05 on log scale, and dotted line (vertical) represents detectable effect at that value.

Simulations of detectable effect sizes for genetic caste determination using both back leg length and centroid size were accomplished using the standard *stats* package and custom scripts in R. For conservative estimates of detectable effect sizes, simulation samples were drawn from a uniform distribution of sizes from the minimum size to the maximum size. Each simulation had 100 replicates, and sampled randomly from a vector of 20 patrilines.


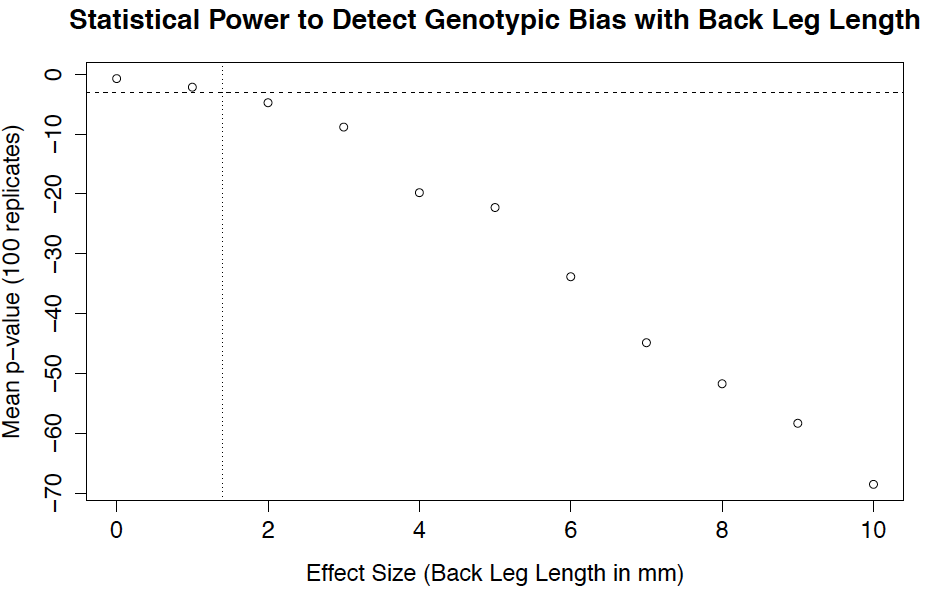


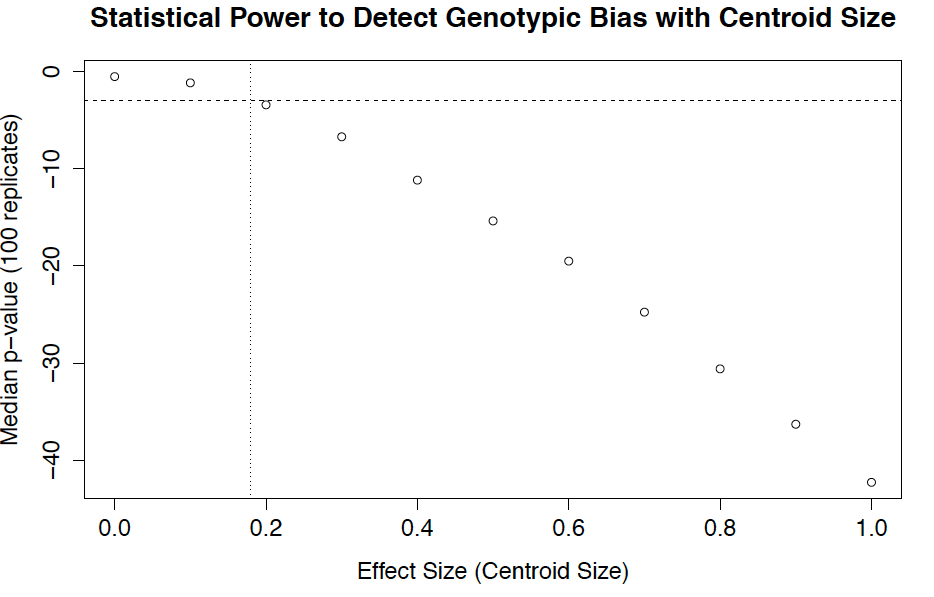


**SI. 5. CAC estimation of static allometry from 48 individuals, with residuals.** Thin plate splines on left plot demonstrate the shape change between the mean form and the form of the lowest (top left) and highest (bottom right) CAC scores as indicated by the arrows. Residual axis 1 of morphological variation in samples plotted against CAC score on right plot.

**SI. 6. Code for data simulations.**

*See R script “simulation_code.R”*

**SI. 7. Code for ML Approach**

*See R script “LRT_test.R”*

**SI. 8. Process for generating simulated morphological data.** All simulated data for estimating detectable effect size of heritable morphological variation followed the process featured below. Simulations in this study were run with 100 individuals to match empirical sample size, however, this is flexible depending on the study.

**
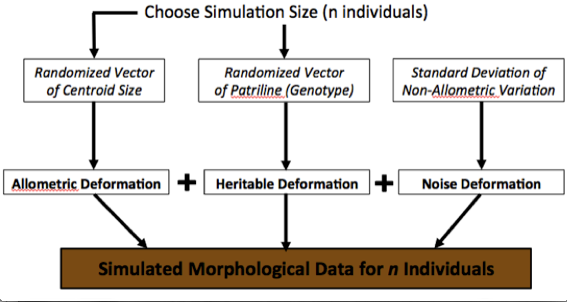
**

**SI.9. PCA-based static allometry from 48 individuals.** Principal components analysis demonstrates the major axis of morphological variation PC1 (42.5% variance), which is highly correlated with centroid size and defines a large component of the static allometry.


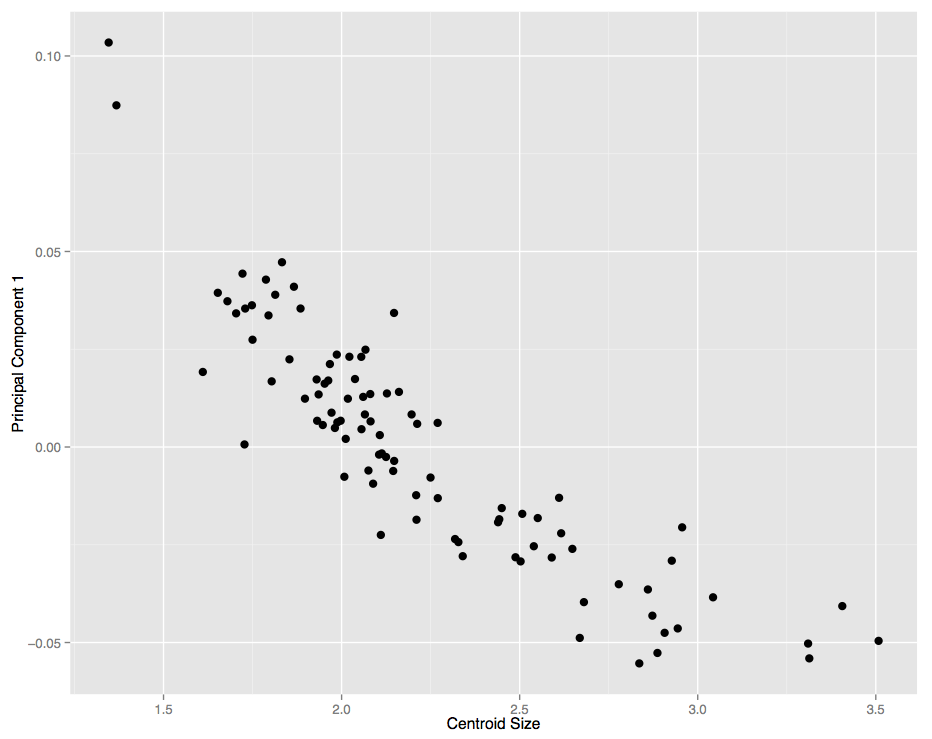


**SI.10. Example replicate dataset demonstrating allometric deformation (AD) along PC1**. Dataset only contains AD, and has no noise deformation (ND) or heritable deformation (HD). Thin plate splines demonstrate the shape change between the mean form and the form of the lowest (top left) and highest (bottom right) PC1 scores, which are generated by simulated centroid sizes.

**SI.11. Plots of principal components (PC1 and PC2) of example replicates for all eight HSD4 treatments from data simulation 2 with thin plate splines demonstrating AD shape change.** Treatments are arranged in columns by orthogonal (HSD4o) and parallel (HDS4p) deformation, and in rows by the mating frequency assigned to the set of replicates. Thin plate splines on each plot demonstrate the shape change between the mean form and the form of the lowest (top left) and highest (bottom right) PC1 scores. Points are colored according to simulated patriline.


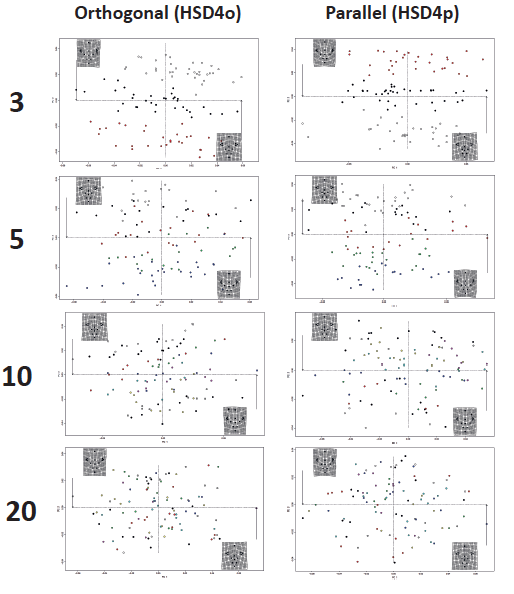


**SI.12. Notes on derivation of Equation 2 and Equation 3.**

*Equation 2* was a choice to model as a multivariate normal distribution, while *Equation 3* derived from the known allometry in the system. Since the known allometry is external to the partitioning of the heritability into heritable and non-heritable components (*Eq. 4*), it was important to account for this with the allometric model (*Eq. 3*). The variance-covariance component (Eq. 4) is incorporated into the basic modeling of principal component scores by setting expectations for variance-covariance among samples.

**SI. 13. Empirical error analysis of morphological variation in technical replicates.**

Pairwise comparisons of differences in centroid size reveals that the scale of technical variation (N = 91, µ = 6.29e-03, σ = 9.9e-03) is close to two orders of magnitude less than the scale of natural variation (N = 4095, µ = 0.49, σ = 0.38) (**Fig. S1**). For the evaluation of differences in shape due to error, the distribution of the sum of squared differences for all landmarks among paired technical replicates was compared to the distribution of the same sum for all pairwise comparisons of natural morphological variation. No individual landmarks contributed significantly more to the technical variation. Results for shape differences mirror the results for size differences (**Fig. S1**), with the scale of technical variation (N = 91, µ = 6.16e-04, σ = 5.53e-04) almost two orders of magnitude less than the scale of natural variation (N = 4095, µ = 1.57e-02, σ = 1.27e-02).

**Fig. S1. The magnitude of measurement error relative to among-individual variation in head size (A) and head shape (B).** In plot A, the left boxplot shows the difference in centroid size between landmark configurations digitized from two photographs of each of 91 specimens (µ = 6.29e-03, σ = 9.9e-03), while the right boxplot shows the difference in centroid size between specimens for the 4,095 pairwise comparisons of the same 91 specimens (µ = 0.49, σ = 0.38). In plot B, the left boxplot shows the difference in shape between landmark configurations digitized from two photographs of each of 91 specimens (N = 91, µ = 6.16e-04, σ = 5.53e-04), while the right boxplot shows the difference in shape between specimens for the 4,095 pairwise comparisons of the same 91 specimens (µ = 1.57e-02, σ = 1.27e-02).
